# Dr. William H. Porter

**Published:** 1861-07

**Authors:** 


DR. WILLIAM H. PORTER, one of the most eminent anatomists, surgeons, and medical practitioners of Dublin, expired in that city on the twenty-eighth of April, after a long, useful, and brilliant career. The immediate cause of his death was probably apoplexy of the brain. We copy the following brief notice of his career from our contemporary, the *London Medical Times and Gazette*, of May fourth:—

"Dr. Porter's University career was a distinguished one, and his classical attainments were of a high order, as was evidenced by his having attained a Scholarship in Trinity College in 1808, when only eighteen years of age. He took the degree of B.A. in the University of Dublin in 1810, and proceeded to the higher degrees of M.A. in 1814 and of M.D. in 1842. In 1814 he became a Licentiate, and in 1817 a Fellow of the Royal College of Surgeons in Ireland. In October, 1836, on the resignation of Dr. Colles, he was elected Professor of the Theory and Practice of Surgery to the College. In 1838 he filled the office of President. He was for forty-one years one of the surgeons to the Meath Hospital and County Dublin Infirmary, and since the death of Sir Philip Crampton, in June, 1858, he was the senior of the six surgeons attached to that institution. Last year, on the death of Dr. Williams, he was chosen to represent the Irish College of Surgeons in the General Council of Medical Education and Registration of the United Kingdom.

"Dr. Porter's contributions to surgical literature have been numerous and highly practical. Among the principal are his 'Observations on the Surgical Pathology of the Larynx and Trachea,' and his 'Observations on the Pathology and Treatment of Aneurism.' In 1836 he contributed to the *Dublin Journal of Medical Science* a paper on Fracture of the Neck of the Femur, an accident which he experienced in his own person not very long before his death. Among his later writings is a series of valuable 'Essays on the Natural History of Syphilis,' to be found in the *Dublin Quarterly Journal of Medical Science.*

"By his decease the surgeoncy to the Meath Hospital, the professorship of surgery in the Royal College of Surgeons in Ireland, and the representation of the College in the General Medical Council became vacant. Thus, although the Medical Act had not yet been three years in operation, the Irish College has twice lost its representative by death. Dr. Porter was seventy-one years of age."

